# Seborrheic Keratosis‐Like Melanoma: Novel Insights Into Clinical, Dermoscopic, and Reflectance Confocal Microscopy Diagnosis of an Atypical Melanoma Variant

**DOI:** 10.1111/srt.70206

**Published:** 2025-07-11

**Authors:** Federico Venturi, Stephano Cedirian, Giulia Veronesi, Biagio Scotti, Carlotta Baraldi, Emi Dika

**Affiliations:** ^1^ Oncologic Dermatology Unit IRCCS Azienda Ospedaliero‐Universitaria Di Bologna Bologna Italy; ^2^ Department of Medical and Surgical Sciences (DIMEC) Alma Mater Studiorum University of Bologna Bologna Italy

**Keywords:** cutaneous melanoma, dermoscopy, reflectance confocal microscopy, seborrheic keratosis

## Abstract

**Background:**

Studies have found that few lesions clinically diagnosed as seborrheic keratosis (SK) revealed Cutaneous melanoma (CM) on histopathology. When CM mimics SK, they are defined as seborrheic keratosis‐like melanoma (SKLM), and a delayed diagnosis and treatment can occur.

**Methods:**

We conducted a retrospective descriptive, monocentric study of the epidemiological, clinical, videodermoscopy (VDS), and reflectance confocal microscopy (RCM) characteristics of histopathologically proven SKLM diagnosed between 2018 and 2024.

**Results:**

The study population consisted of 60 patients: 44 males (73.3%) and 16 females (26.7%) with lesions located in 73.3% of cases on the trunk. Concerning histological type, superficial spreading melanoma was the more frequent (86.7%). More than 50% of tumors had a Breslow thickness <0.8 mm. The findings from the VDS examination using the revised Argenziano Seven Point Checklist revealed that 100% of cases presented at least one dermoscopic melanoma‐specific criterion and, for this reason, had an indication for surgical excision. Typical RCM patterns associated with melanoma diagnosis were observed, including irregular honeycomb/cobblestone pattern (82.1%), irregular DEJ nests (78.6%), dermal inflammation (53.6%), irregular dermal nests (53.6%), dendritic cells in sheets/tangled lines (50%), and atypical round cells (39.3%).

**Conclusion:**

Our study provides valuable insights into the dermoscopic, RCM, and histological features of SKLM based on the largest monocentric cohort. The distinctive dermoscopic patterns, along with the confocal features, aid in the differentiation from other pigmented lesions.

## 1 Introduction

Studies have found that few lesions clinically diagnosed as seborrheic keratosis (SK) revealed Cutaneous melanoma (CM) on histopathology. When CM mimics SK, they are defined as seborrheic keratosis‐like melanoma (SKLM), and a delayed diagnosis and treatment can occur [1]. Other benign tumors, as well as potentially pre‐cancerous or malignant conditions, may resemble the characteristic clinical features of SK, and a prompt recognition of such tumors using noninvasive diagnostic tools is mandatory to reduce misdiagnosis and facilitate clinicians in the management process [2, 3, 4, 5, 6]. The role of dermoscopy, videodermoscopy (VDS), and reflectance confocal microscopy (RCM) in enhancing diagnostic accuracy for cases of SKLM has been explored in limited studies [6, 7, 8, 9, 10]. Our retrospective analysis encompassed clinical, dermoscopic, and RCM characteristics of SKLM to identify specific features aiding clinicians in differentiating SKLM from SK using non‐invasive diagnostic techniques.

## 2 Materials and Methods

We conducted a retrospective descriptive, monocentric study of the epidemiological, clinical, VDS, and RCM characteristics of histopathologically proven SKLM. We collected the histopathologic charts of CM diagnosed between January 2018 and November 2024, presenting on the clinical query the diagnosis of suspected SK from the Oncologic Dermatology Unit, IRCCS Azienda Ospedaliero‐Universitaria of Bologna, Policlinico of Sant'Orsola. We obtained clinical and pathologic data from the charts for each patient. Sixty SKLM cases were selected. Patients were informed about the study, and a written informed consent for publication of the photographs used in this manuscript was obtained. Clinical and VDS images (×20 and ×40 magnification) obtained using FotoFinderMedicam 800HD (FotoFinder, Germany) were reviewed by two dermatologists with expertise in the field to confirm the definition of SKLM (G.V. and C.B.). We leveraged the Argenziano revised Seven Point Checklist to define standardized dermoscopic characteristics [11]. The seven‐point checklist is one of the established methods to diagnose melanoma dermoscopically. This algorithm is based on the recognition of seven melanoma‐specific criteria, each of which is classified as major or minor. The presence of one of the three major melanoma‐specific dermoscopic features (Atypical network, Blue‐white veil, and atypical vascular pattern) is scored as 2 points, and the presence of one of the four minor features (irregular dots/globules, irregular blotches, and regression structures) is scored as 1 point. Under the revised seven‐point algorithm, excision is recommended if the total score is ≥1, with a sensitivity of 87.8% and a specificity of 74.5%. Among all lesions, 28 were referred for RCM evaluation. RCM investigation was performed with a Vivascope (MAVIG GmbH, Munich, Lucid‐Tech Inc., Henrietta, NY, USA) microscopy. Each skin lesions were systematically evaluated by RCM: one X–Y horizontal mapping (4 × 4‐mm mosaic) was performed at each epidermal layer beginning with the stratum corneum through the entire epidermis and until the papillary dermis (maximal depth of imaging: 200–250 µm). Lesion images were evaluated by two expert dermatologists in the field (F.V. and E.D.) according to eight established RCM melanoma criteria (Honeycomb/cobblestone pattern, DEJ pattern, ringed meshwork, dendritic cells, atypical round cells, DEJ nests, dermal nests, inflammation, and cerebriform nests) previously validated [9]. Finally, hematoxylin‐eosin slides of the 60 cases were independently reviewed by two pathologists (C.B. and C.M.).

## 3 Results

### 3.1 Epidemiological Data

The study population consisted of 60 patients with a histologically confirmed diagnosis of SKLM, including 44 males (73.3%) and 16 females (26.7%). Their age at presentation was <50 years old in 50% of cases, whereas among patients ≥50 years old, the majority were placed in the range 50–70 (26.7%). The anatomical sites were as follows: 73.3% of SKLM were located on the trunk, 20% on the limbs, and 6.7% on the head and neck. Concerning histological type, superficial spreading melanoma was found in 52 cases (86.7%), nodular melanoma in 6 cases (10%), and nevoid melanoma in 2 cases (3.3%). 40 cases displayed a radial growth phase (66.7%), whereas 20 cases (33.3%) displayed a vertical growth phase. Melanoma in situ was reported in 6.7% of cases. Regarding invasive CM, 32 cases (53.3%) had a thickness <0.8 mm, while 28 (46.7%) had a thickness of ≥0.8 mm. Staging according to the AJCC eighth edition demonstrated that 6.7% were classified as pTis, 50% as pT1a, 10% as pT1b, 13.3% as pT2, 13.3% as pT3, and 6.7% as pT4 (Table 1).

**TABLE 1:** Epidemiologic and histopathological features of SKLM according to the 2018 WHO classification of skin tumors and the eighth edition of the AJCC cancer staging manual melanoma [19, 20].

Clinical and pathologic data	*N* (60)	%
Age–years		
<50	30	50
50–70	16	26.7
≥70	14	23.3
Gender		
Female	16	26.7
Male	44	73.3
Location		
Trunk	44	73.3
Head and neck	4	6.7
Limbs	12	20
Histopathologic subtype		
Superficial spreading melanoma	52	86.7
Nodular melanoma	6	10
Nevoid melanoma	2	3.3
Growth pattern		
Radial	40	66.7
Vertical	20	33.3
Breslow thickness		
<0.8 mm	32	53.3
≥0.8 mm	28	46.7
Regression		
Present	18	30
Absent	42	70
Ulceration		
Present	4	6.7
Absent	56	93.3
Stage (according to AJCC 8th ed.)		
pTis	4	6.7
pT1a	30	50
pT1b	6	10
pT2	8	13.3
pT3	8	13.3
pT4	4	6.7

### 3.2 Dermoscopic and RCM Features

**FIGURE 1:**
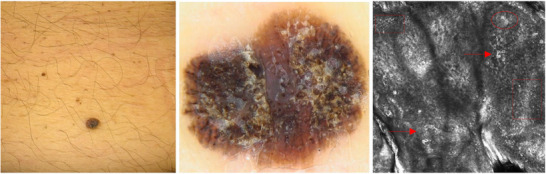
(A, B, C) Clinical, dermoscopic, and confocal presentation of SKLM on the back of a 55‐year‐old patient. At clinical examination, SKLM presented as a hyperpigmented, hyperkeratotic nodule (A). Dermoscopic examination revealed an irregular pigmentation, blue‐white veil, and irregular streaks (B). Reflectance confocal microscopy of SKLM displayed irregular DEJ nests (red circle), dendritic cells (red box), and atypical round cells (arrows) (C).

**FIGURE 2:**
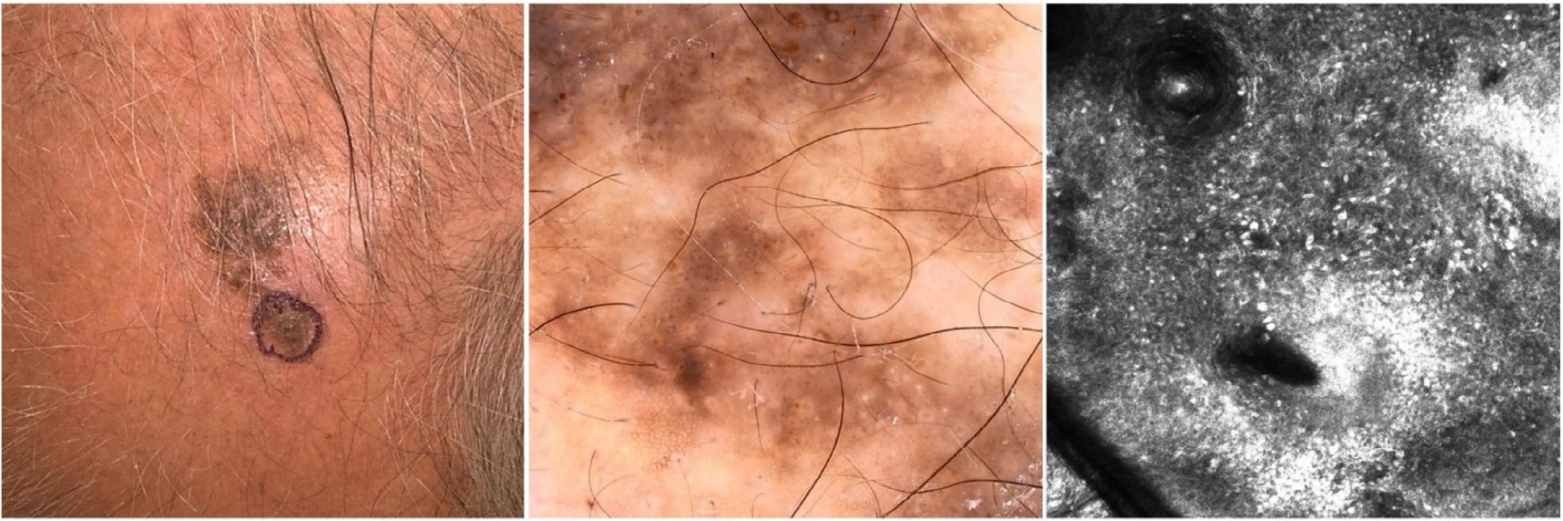
(A, B, C) Clinical, dermoscopic, and confocal presentation of SKLM on the scalp of a 68‐year‐old patient. At clinical examination, SKLM presented as a flat pigmented macule with irregular border and perifollicular pigmentation (A). Dermoscopic examination of the lower part of the lesion revealed asymmetry of structures, atypical pigmentary network, atypical globules and irregular dots, areas with irregular homogeneous pigmentation and comedo‐like openings (B). Reflectance confocal microscopy of SKLM displayed the epidermal disarray with atypical cells surrounding the follicular openings (C).

The findings from the VDS examination using the revised Argenziano Seven Point Checklist revealed that irregular pigmentation appeared in 52 cases, constituting approximately 86.7% of the total cases examined. An atypical pigment network was found in 30 lesions (50%), while atypical vascular patterns were found in 18 patients (30%). Additionally, irregular streaks were identified in 14 cases (23.3%), and regression structures in 20 lesions (33.3%). Furthermore, blue‐whitish veil was described in 12 tumors (20%), and irregular dots/globules were found in 8 cases (13.3%). Moreover, the utility of the acknowledged algorithm is to diagnose melanoma dermoscopically. In our series, 100% of cases presented at least one criterion and for this reason had an indication for surgical excision (Figures 1A,B and 2A,B).

In most lesions, typical RCM patterns associated with melanoma diagnosis were observed, including irregular honeycomb/cobblestone pattern (82.1%), irregular DEJ nests (78.6%), dermal inflammation (53.6%), irregular dermal nests (53.6%), dendritic cells in sheets/ tangled lines (50%), and atypical round cells (39.3%) (Figures 1C and 2C). Results are fully displayed in Table 2.

**TABLE 2:** Dermoscopic and RCM features of SKLM.

Dermoscopic features	*N* (60)	%
Atypical pigment network	30	50
Blue‐white veil	12	20
Atypical vascular pattern	18	30
Irregular streaks	14	23.3
Irregular pigmentation	52	86.7
Irregular dots/globules	8	13.3
Regression structures	20	33.3

**RCM features**	***N* (28)**	**%**
Honeycomb/cobblestone pattern		
Regular	5	17.9
Irregular	23	82.1
Dermo‐epidermal junction pattern		
Ringed	3	10.7
Meshwork	13	46.4
Clod	1	3.6
Combined	1	3.6
Non specific	10	35.7
Dendritic cells in sheets/tangled lines	14	50
Atypical round cells	11	39.3
Dermo‐epidermal junction nests		
Regular	5	17.9
Irregular	22	78.6
Dermal nests		
Regular	6	21.4
Irregular	15	53.6
Inflammation	15	53.6
Cerebriform nests	6	21.4

*Note*: Dermoscopic examination was performed according to the Argenziano revised seven‐point checklist.

## 4 Discussion

As a matter of fact, SKLM represents a rare occurrence in our clinical practice and requires the careful evaluation of dermatologists and dermatopathologists with expertise in the field. The association of SK features in CM has been thought to represent a collision phenomenon, while others have suggested induction of SK‐like features in the epithelial component by the melanocytes [12]. The epidermal overgrowth could be induced by a network between melanocytes and keratinocytes involving the epidermal growth factor receptor (EGFR), the fibroblast growth factor receptor (specifically FGFR3), and other pathways [13, 14, 15, 16]. For a better comprehension of such a phenomenon, and to characterize SKLM, we evaluated the main dermoscopic RCM and histopathological features of these tumors to identify the presence of possible clues for their identification. To date, only a few papers have investigated such aspects [6, 9, 17, 18]. A previous study described the presence of dermoscopic melanoma features only in 82% of cases [6]. This underlines the difficulty in diagnosing this clinical subtype of melanoma with the naked eye. In our series, using the revised 7‐point checklist scoring system, 100% of cases presented at least one dermoscopic melanoma‐specific criterion and, for this reason, had an indication for surgical excision, further validating such a great algorithm. Moreover, we performed RCM on 28 difficult lesions. Surprisingly, only a few studies investigated SK‐like tumors’ RCM features. A possible explanation is derived from its limited application to this kind of lesion due to the presence of superficial hyperkeratosis. A recent study, based on smaller multicentric cohorts of 19 cases of SKLM, described the presence of an irregular honeycomb pattern in most cases combined with dendritic cells in sheets or tangled lines, atypical round cells, and irregular dermal nests [9]. Our data partially confirm previous reports, with a lower percentage of the irregular honeycomb/cobblestone pattern (82.1% vs. 94.7%) and dermal inflammation (53.6% vs. 89.5). Specific melanoma features were described in all cases, enabling a clear diagnostic assumption of melanoma. Histopathologically, most lesions revealed superficial spreading melanoma subtype (86.7%) with a low incidence of regression (30%) or ulceration (6.7%). More than 50% of the tumors were classified as ≤pT1a. Moreover, in a small percentage of tumors, it was possible to identify a specific pattern of SKLM defined by the presence of a dermal and expansive melanomatous component in a vertical growth phase mixed with an SK‐like component of acanthotic‐type (large pseudohorn cyst, exophytic and papillomatous appearance with hyperkeratosis, focal acanthosis and epidermal hyperplasia).

## 5 Conclusion

In conclusion, our study provides valuable insights into the dermoscopic, RCM, and histological features of SKLM based on the largest monocentric cohort. The distinctive dermoscopic patterns, along with the confocal features, aid in the differentiation from other pigmented lesions. However, distinguishing SKLM remains a diagnostic challenge both for clinicians and pathologists.

## Ethics Statement

The study and data acquisition protocol were approved by the Local Ethics Committee, and it was carried out in accordance with the Declaration of Helsinki.

## Consent

Patients were informed about the use of their clinical information according to the Declaration of Helsinki principles and photos for a publication intent. Written informed consent was obtained from the patients for publication of the details of their medical case and any accompanying images.

## Conflicts of Interest

The authors declare no conflicts of interest.

## Data Availability

The data used during the current study are available from the corresponding author on reasonable request.
